# The Urban River Syndrome: Achieving Sustainability Against a Backdrop of Accelerating Change

**DOI:** 10.3390/ijerph18126406

**Published:** 2021-06-13

**Authors:** Martin Richardson, Mikhail Soloviev

**Affiliations:** Department of Biological Sciences, Royal Holloway University of London, Egham TW20 0EX, UK

**Keywords:** urban river syndrome, urban stream syndrome, pollution, sustainability, ecosystem, adaptation, anthropogenic changes, the Thames, restoration

## Abstract

Human activities have been affecting rivers and other natural systems for millennia. Anthropogenic changes to rivers over the last few centuries led to the accelerating state of decline of coastal and estuarine regions globally. Urban rivers are parts of larger catchment ecosystems, which in turn form parts of wider nested, interconnected systems. Accurate modelling of urban rivers may not be possible because of the complex multisystem interactions operating concurrently and over different spatial and temporal scales. This paper overviews urban river syndrome, the accelerating deterioration of urban river ecology, and outlines growing conservation challenges of river restoration projects. This paper also reviews the river Thames, which is a typical urban river that suffers from growing anthropogenic effects and thus represents all urban rivers of similar type. A particular emphasis is made on ecosystem adaptation, widespread extinctions and the proliferation of non-native species in the urban Thames. This research emphasizes the need for a holistic systems approach to urban river restoration.

## 1. Introduction

Anthropogenic changes to rivers have been apparent for at least two centuries [1], and despite sporadic improvements [2], coastal and estuarine regions are in an accelerating state of decline. Work is needed to catch up with a historical deficit in terms of accumulated pollution, waste and other damage. There is a long history associated with the study of rivers (Table 1). For example, stream invertebrates have been used as indicators of water quality since the 19th century, e.g., the Macroinvertebrate Community Index in New Zealand. Theories to predict ecological patterns associated with features of river morphology have been widely employed for many years [3,4]. However, some such theories assume pristine baseline conditions, which are no longer appropriate. Nonetheless, due to their historical value, these theories are continually updated and are in use at the current time.

**Figure 1:**
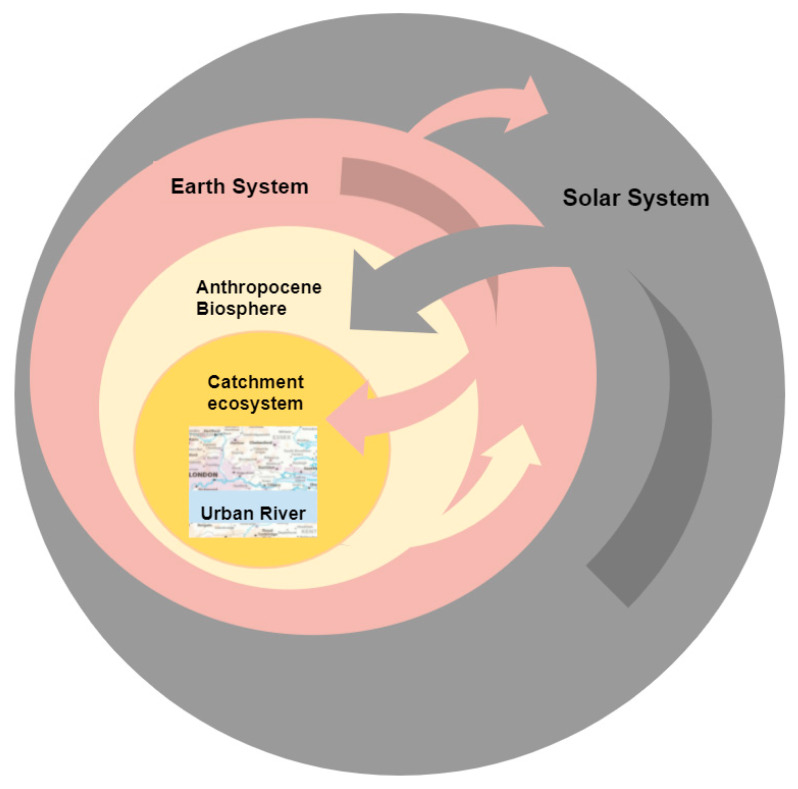
Interactions between nested, interconnected systems operating over different spatial and temporal scales. The solar system affects tides, causes mutations in DNA through cosmic radiation, mass extinctions and changes in geomorphology through meteor impacts, and solar energy powers much of life on earth. The Earth system returns heat to space in maintaining equilibrium and affects the biosphere in multiple ways, including climate change. Feedbacks from the biosphere influence the Earth system and return anthropogenic pollution.

Urban rivers are one component of catchment systems. Though not usually studied together [7], system changes are connected and coactive. They operate at different levels (Figure 1) and at different rates, which means they can give rise to unexpected outcomes in the form of ‘surprises’ [8] or ‘shocks’ and even ecosystem collapse, which are recognised features of dynamical systems behaviour in general. Close observation, monitoring and assessment, is thus increasingly important. Whilst generally problematic, some changes may be beneficial, at least temporarily for certain species, e.g., invasive species, which may thrive as environmental parameters (e.g., temperature) transition through their preferred range or niche and a population irruption occurs. Other changes interact antagonistically to reduce their individual effect, but this is happening against a backdrop of overall decline. River catchments are affected by connections to the wider Earth system and beyond along a gradient of risk in geologic time pertaining to landslides, earthquakes, and other hazards. Individual stressors vary in temporal and spatial extent, they drive ecosystem response and inform adaptation as well as the potential longevity of restoration work. Their persistence and the rate at which they are changing, both when speeding up and also when slowing down, are often overlooked. Just like rivers, which over millennia sculpt intricate paths across a landscape, ecosystems will change, adapt and re-assemble. Such processes may take far longer than several decades and therefore could not be observed in their entirety over the course of a human lifetime, a phenomenon known as ‘shifting baseline syndrome’ (Papworth, 2009). In this respect, it is worth considering the changes wrought through the building of dams, for example, over the previous few centuries.

Accurate modelling of a complex system such as a river catchment may not be possible. Many components of such a system may be unknown, poorly understood or inaccessible to observation, e.g., be underwater or below ground. Knock-on effects generated by such components, the so-called co-benefits and trade-offs, may be either positive or negative. This chain of events continues *ad infinitum*, meaning that a degree of abstraction is required. However, these difficulties, though seldom recognised, have always applied to the limits of knowledge of ecosystems. This has made correlating multiple, individual studies particularly difficult since the relative importance of the effects is unclear. It is not always possible to make a connection between studies, especially when different sets of environmental variables are used. An isomorphic issue has recently been addressed by the Coupled Model Intercomparison Project (CMIP6) through adoption of a standardised set of experiments and other forms of rationalisation [9]. However, at the present time, it is inevitable that different ecosystem studies, especially at the detailed level, will produce different results. Accelerating rates of change of key stressors [10] threaten to render much of what has been learnt about ecosystems obsolete. Further, change is far outpacing the ability of many species to disperse or adapt, making it probable we are entering an as yet unrecognised chaotic epoch as ecosystems destabilise further, leading to the sixth major extinction [11].

Pollution is a fundamental problem. Urban rivers will continue to decline until comprehensive solutions are developed and implemented. Urban run-off [12,13,14] has sublethal effects but can kill fish at all life stages: eggs, larvae, juveniles and adult [15]. Degraded urban waterways have higher levels of parasitism in all taxa [16], many of them invasive, e.g., *Anguillicoloides crassus* in eels. Chemicals are released into groundwater and surface waters through multiple routes. Animal treatments for livestock, which may promote parasitism through development of resistance, and fertilizers used in agriculture are washed into rivers with soil as run-off from fields [17]. Many can also be deposited directly by the wind (atmospheric deposition). Some chemicals, including nicotinoid pesticides, are water-soluble, and that makes them even more mobile [17]. Manure from grazing animals in fields may aggravate organic pollution in the uplands and lowlands. Slurry from cattle and solid waste from pigs, chickens and other animals contain antibiotics and antimicrobial-resistant bacteria (ARB) and are often stored close to bodies of water. Warming waters with reduced oxygen content and enriched with nutrients make surface water eutrophic and anoxic, which aggravates these issues, especially in the lower reaches of rivers and estuaries. In most major rivers, a nutrient-rich, toxic plume extends into the ocean, where it can stimulate harmful algal blooms, further aided by increasing temperatures. Associated anoxic conditions, ‘dead zones’, in combination with generally reduced oxygen affect significant areas of the oceans, although recent studies indicate that they may not become widely distributed throughout oceans globally [18,19].

**Figure 2:**
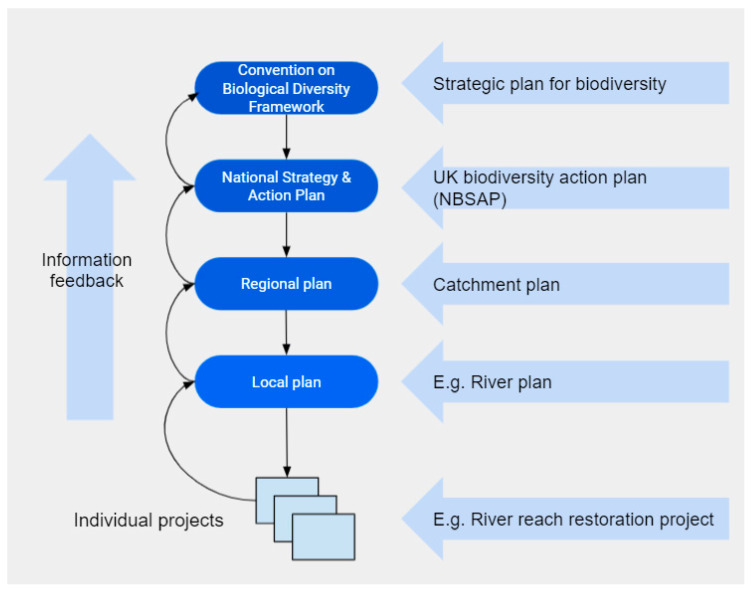
Hierarchical framework for coordinated river restoration work at global to local scale. NBSAP refers to the National Biodiversity Strategies and Action Plans described in Article 6 of the United Nations Convention on Biological Diversity (CBD).

Habitat stewardship and species husbandry have been conducted by indigenous peoples for millennia [20,21,22]. To achieve a sustainable future, these will need to be incorporated as a central tenet of the Anthropocene together with a radical change in environmental hygiene in general. To be socially and culturally relevant and therefore to progress, restoration must include local and regional stakeholders and any work prioritized and justified through valuation within a global framework (Figure 2). River restoration projects are typically conducted over a relatively short period of time (one or two years) and often with little follow-up monitoring. Even well-funded, long-term restoration projects may be overshadowed and overcome by climate change and other stressors that were not foreseen or properly considered beforehand, as was the case with salmon habitat restoration in the United States [23]. Such changes and stressors might override ecosystem restoration and therefore must be assessed and anticipated. Therefore, a wide variety of related factors should be considered, for example, through modelling, to formulate a hypothesis and a model (albeit imperfect) as to how these might interact with each other, influence system function and affect the outcomes. Even small changes may render restoration work obsolete, and there is increasing potential for highly destructive ‘extreme events’, e.g., major flooding, to cause extensive damage in a typical, heavily engineered urban river catchment. Accumulation of pollution, species loss, the potential for disease transmission and flooding are of particular concern. Further work is necessary to address these compounding problems, to improve overall resilience and reduce the possibility of further shocks. Achieving sustainability while starting from a condition of accumulated deficit against a backdrop of accelerating change will require continuous environmental stewardship. It will mean making hard-earned incremental improvements [24] within a coordinated, over-arching catchment plan, which can be adapted in response to system behaviour. Just as ‘continuous change’ [25] became the mantra of business in the 1990s, ‘continuous adaptation’ might be a suitable motto for environmental work in the Anthropocene.

## 2. The Urban River Syndrome—Ecosystems Previously Transformed

Surface water impairment, especially eutrophication and water quality, has been studied since the late 1960s. Overall degradation has been understood to be progressing for at least 60 years, but assessment and improvements have not kept up. The gradual, generally monotonic decline produced by the urban river (stream) syndrome (URS) [26,27] can be used to describe newer or previously unknown types of river ecosystems degradation, such as that resulting from additional waste materials such as plastics [28] and chemicals, including pharmaceuticals [29,30] and narcotics [31,32], in the sediment, soil and water. Changes in temperature regimes and patterns of rainfall, and ecosystem modifications, e.g., fisheries augmentation and species range shifts, are also not included. However, not all such effects are negative. For example, increased UV light can promote destruction of xenobiotics and pathogens. The positive effect of turbidity on photolysis (by means of increasing diffusion of light through water) contributes to purification in wetlands, which was recognised long ago. Wildlife switching to human food items alters food webs and can promote unnatural growth, but may also alter behaviour and promote disease in animals [33,34].

Large urban rivers are now characterised by shipping and container ports, power stations, increased boat traffic, accelerating rates of non-native species settlement and light and noise pollution. Inadequate sewage treatment and increased fine sediment and effluent presage health concerns with regard to the possibility of zoonoses and outbreaks of novel disease. The growing size of cities with associated increases in storm run-off from roads and combined sewer systems, reduced oxygen content, erosion of topsoil from agricultural areas [35] and the effects of nutrient, pesticide and salt pollution are all established concurrent synergistic features. Rubbish and toxin accumulation in sediment with anoxic plumes and harmful algal blooms in the estuary are fundamental for a description of an urban river such as the Thames (Table 2). 

While range shifts are now understood to be occurring in many species [65], more nuanced ecological impacts from increasing light and sound pollution have far-reaching effects on both terrestrial and aquatic animals. In extreme instances, pile-driving used for installation of river revetments or other construction can cause physical harm and complex behavioural changes such as avoidance and reduced vocalization [54,66,67]. Urban birds now sing differently, employing complex strategies to compensate for increased ambient levels of noise [68], but it is reasonable to believe that there are wider effects on the ecosystem generally. Fish such as cod, *Gadus morhua*, use sound for communication in agonistic displays and during courtship [66,69,70]. Changes to river structure from dams and modified flow as well as boat and shipping traffic affect the ‘soundscape’ [71], the ambient underwater sound profile, changing behaviour in fish. Some species have adapted to this and increase predation success by taking advantage of confusion in prey [72]. Similarly, light pollution affects bats and insects, with mayflies being attracted to lamps, for example [73]. Many species of insects (e.g., fireflies) and fish have evolved complex signalling through bioluminescence. Night-time light pollution can affect diel vertical migration of plankton in the water column and other aquatic species, including many salmonids that synchronise spawning in accordance with lunar cycles [74], which are also vulnerable to disruption.

In cities, rivers have been historically used to transport waste. Wastewater (before treatment) consists mainly of water with a small amount of solid material, most of which is organic and includes food waste, faecal matter, urine and soaps. Two metrics are frequently associated with wastewater treatment. One is chemical oxygen demand (COD), which is a theoretical measure of the oxygen requirement for breaking down organic matter, even though materials such as fats are not completely broken down. Bacterial oxygen demand (BOD) uses bacterial digestion to assess potential environmental impact, but the test would need to continue for an infinitely long time to be accurate. Therefore, BOD generally underestimates the oxygen requirement for material to reach a stable state of degradation. Sewage treatment plants (STPs) use a lot of electricity to circulate and oxygenate ponds and enable aerobic bacteria to break down waste, producing greenhouse gas (GHG) emissions. Reducing oxygen levels saves energy but can increase production of NO_2_—a more powerful, shorter-lived GHG than CO_2_, which is otherwise produced. Storm water flows are increasing because of climate change [75], and many STPs are not designed to accommodate the increase in run-off, and despite the water being improved after treatment, there are still many contaminants that are not removed in the current processes used.

It is widely recognised that there are thousands of man-made chemicals circulating in the environment—a major feature of the Anthropocene [76,77]. The biological effects of most are understudied since the majority of research into toxicity is focused on a relatively small number of familiar compounds [78,79]. Sometimes these are used as models from which wider inferences and conclusions are made without investigating analogous yet non-identical compounds, and combinations are rarely considered despite synergistic effects being well known for many. Endocrine disruptors such as heavy metals and synthetic oestrogens cause intersex in aquatic animals [80]. Pharmaceuticals, including antibiotics from farm use or waste water, alter microbial communities [81,82], play a role in the development of antibiotic resistant-strains of bacteria such as *Staphlococcus aureus* (MRSA) and are capable of affecting human health and presenting a wider escalating problem [83,84]. Phosphorous and heavy metals such as cadmium and mercury accumulate in river banks and benthic sediment [85] and can be remobilized through erosion, dredging, boat traffic, riverside works or increased river flows. These subsequently accumulate in fish, birds, pinnipeds and cetaceans and magnify as they progress up the food chain [86], which results in contaminant loads that affect reproduction and behaviour. However, loss of fertility is a concern even at lower trophic levels [87].

Chemical pollution may impact the role of semiochemicals, affecting interactions between species and conspecifics, particularly those occurring over longer distances, such as herbivory defence in plants and prey availability in animals [57]. Olfactory reception plays an important role in navigation in fish and birds [88], and pheromones are important during reproduction in fish, both internally in terms of reproductive function and also externally to aid synchrony [89]. There are other types of ecologically important chemical signals used by plants and animals, for example, attractants, repellents and kairomones. Some of these are beneficial to the host and detrimental to other species, and vice versa, indicating a complex sensory landscape. However, individual compounds have been found to affect behaviour through chemical pathways. Rats, for example, undergo profound behavioural changes when exposed to certain chemicals, even at low concentrations [56]. Low-level exposure to copper in juvenile salmon renders them more vulnerable to predators [90]. Psychoactive drugs are specifically designed to work at low concentrations and these are now ubiquitous in urban rivers, affecting behaviour [91].

**Figure 3:**
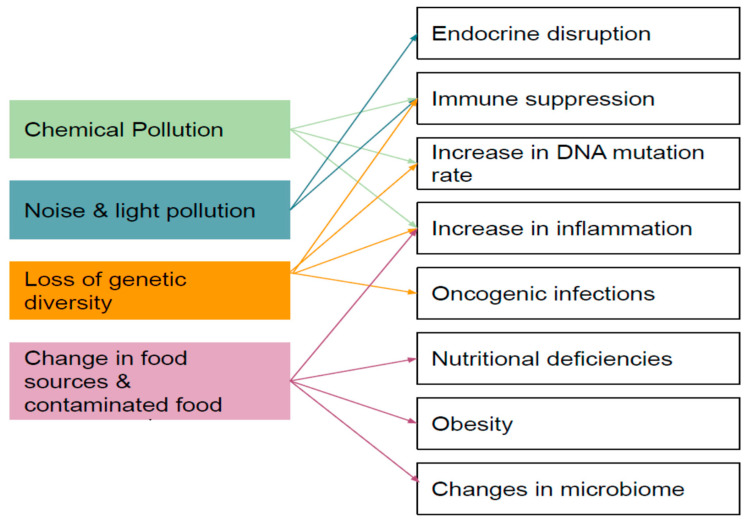
Pathways from effects caused by human activity that may promote disease in wild animals [33].

A sometimes overlooked aspect of change is the increase of cancers in animals [34,92]. Heavy metals and other xenobiotics, including those released by plastics following ingestion, can induce cancer. There are multiple other ways humans increase the prevalence of cancer in animals (Figure 3). Wild trout, once shy, have changed their behaviour and take bread from humans, which promotes growth and accumulation of fat. This leads in turn to accumulation of lipophilic xenobiotics. Even pollution-tolerant species like catfish can develop tumours [47], though little is known about the effects of neoplasia on mortality generally. Another feature of urban rivers is intersex in fish, gastropods and molluscs, which frequently occurs because of parasites [93] or endocrine disruptors such as synthetic hormones in wastewater effluent or industrial activities [94]. Feminisation of male roach, *Rutilus rutilus*, occurs widely in English rivers [95]. Many aquatic species change sex as a feature of their lifecycle and are sensitive to changes in environmental hormone levels, which can result in populations becoming less viable [96].

Physical changes to urban rivers and streams over the past several decades show shrinking dendritic topology and increasing patchiness due to modification and climate change, with reductions expected to increase further [97]. Despite clean-up of industrial point source pollution, there has been a transformation in appearance of freshwater bodies over a period of several decades, with reduction in shoreline and benthic macrophytes due to reduction of the euphotic zone and silt smothering of the substrate. Chalk streams in the UK have a perpetual layer of brown algae covering the substrate. Similarly, there have been changes in species composition with increasing homogeneity, transformation of microbial communities and declines in native fish, insect and bird populations [98,99,100]. Cold water species and river flies have seen major declines [101,102]. In the UK, the fall in river fly numbers has been detailed in the 2015 Riverfly Census compiled by Salmon and Trout Conservation UK [101]. Phenotypic and eco-evolutionary change in native fish and plants is widespread [103], the phenomenon exacerbated in urban rivers, with humans becoming one of the primary drivers of evolution [104]. River flies that spend most or part of their lifecycle in aquatic environments and other invertebrates are undergoing large reductions, in part because of agricultural insecticide pollution [105]. Herbicide use, e.g., glyphosate, has transformed grassland and riparian habitat especially in urban settings. Weed treatments can drastically alter a river’s insect populations through complicated mechanisms, which may be difficult to foresee. Genetically modified crops, as used in some countries, might have similarly unpredictable outcomes for insects, a key component of the riverine food web. For example, the expansion of transgenic corn in Iowa in the United States resulted in dramatic decline of the Monarch butterfly, *Danaus plexippus*, through deposition of toxic pollen on leaves of the Milkweed plant, *Asclepias syriaca*, on which the larvae feed [106]. That and other similar multifaceted interactions, many of which may yet be unknown, will most probably be occurring on a larger scale in and around rivers, thus altering communities of fish, insects, birds and mammals. Many commonly used agricultural chemicals are water-soluble, thereby having direct impacts on aquatic plants and animals through run-off and atmospheric deposition when blown as dust from fields into waterways.

Trees are an important component of riparian habitat. Their root systems can increase bank stability, and the shade they provide helps maintain lower water temperatures, which can be beneficial to many aquatic species as climate warms. While improvements are being made to riparian vegetation and margins between roads and the river in some locations, the overall condition is impaired through climate change (drought and flooding), die-back, temperature changes and non-native diseases such as root and collar rot in alder, *Alnus glutinosa*, caused by *Phytophthora alni*. Several other non-native pathogens are affecting riparian vegetation (Table 3), and more may arrive in the UK in future, such as *Ceratocystis platani* fungus, affecting plane trees. While this pathogen has not been reported in the UK, the fungus affects trees along river corridors in other European countries, where it is particularly problematic due to water-borne transmission [107]. The insect vector for this pathogen, a beetle, *Platypus cylindrus*, acts as a pioneer, facilitating further attacks by other species. It was once rare in the UK but is now common, paving the way for rapid spread upon arrival [108]. Additional stressors on riparian habitat include excess nutrients and pollution in the soil and water, which can impair growth by interfering with the mycorrhizal fungi, replacing fungi that provide nutrients in return for carbon with parasitic species.

## 3. Ecosystem Adaptation and Non-Native Species in the Urban Thames 

**Figure 4:**
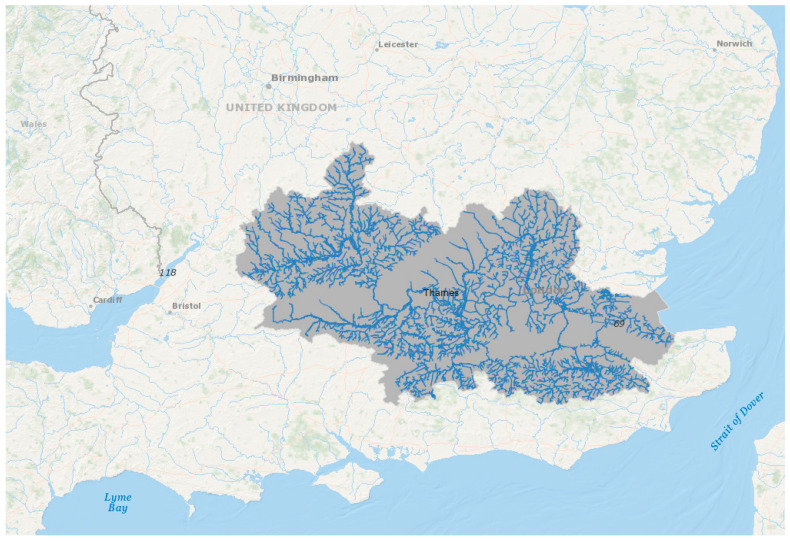
The Thames catchment. Image created in ESRI ArcGIS using data from the UK Ordnance Survey and European Environment Agency.

The Thames is a typical urban river that suffers from growing anthropogenic effects, as is the case with all rivers of similar type. It is a highly modified, fragmented river (Figure 4) with a long history of exploitation of fisheries, disposal of wastes and transport. It provides some of the largest green space in London, helps to mediate the heat island effect in the city and offers extensive opportunities for recreation. The urban Thames suffered a major decline in the first half of the 19th century due to rapid increases in population in London as well as other towns along the river, resulting in increased discharges to the Thames and tributaries. As a result, oxygen content of the water became severely reduced. The opening of the London sewer system in 1870 improved the situation temporarily, but the acute oxygen depletion problem returned during the first half of the 20th century as the system became overwhelmed. Stretches of the tidal Thames again turned anoxic with widespread hypoxia and anoxic sediment, resulting in a gross depletion of aquatic life. Additional improvements to the sewer network in the second half of the 20th century, termed ‘sanitisation’, resulted in amelioration of these harsh environmental conditions with a concomitant re-use of the river by endemic UK North Sea coastal species. Recent remedial action around point source pollution has generally improved some aspects of water quality, although pollution events still occur regularly; continuing diffuse pollution is another growing problem. Migratory native species that use the entire catchment, such as salmon, have not re-colonised the river in significant numbers and populations have fluctuated, with many opportunistic, non-native species becoming permanently established and new species immigrating at an accelerating pace. Attempts to restore Atlantic salmon, *Salmo salar*, through stock augmentation and improved river connectivity have not met with success, and although salmon do occur in the Thames, they originate from other rivers [109]. Overall, the tidal community is now adapted to polluted, low-oxygen conditions with high turbidity and silt smothering the benthos. 

The overall decline in condition and associated biodiversity loss in the Thames, particularly in terms of reduced abundance, have gone largely unreported until recently. However, it is to be expected that biodiversity loss is generally manifested as a decline in abundance with highly reduced populations rather than complete extirpation. Stock augmentation and hybridisation cause reduction in genetic biodiversity [110], so the overall decline is chronic rather than acute. Although serious pollution events and mass die-offs do occur on occasion, these do not usually affect the entire river, and many species use different areas of habitat at different times of year and varying stages of their lifecycle. Extinction is no doubt a continuing process for many native species and this will persist until overall conditions start to improve, or until they are able to translocate to a more suitable habitat. Therefore, complete, local (e.g., within the tidal Thames) extirpation is a long-term process.

A typical feature of urban rivers is an increasing number of non-native species [111]. Much like other similar river systems globally, the Thames catchment is home to a large and increasing number of non-native species [37]. Some are presently—or have previously been—invasive. They include ornamental garden plants like rhododendron, which grows wild in many areas of the UK, fish such as goldfish, *Carassius auratus auratus*, which can grow large and create hybrids, and birds, including the ring-necked parakeet, *Psittacula krameria*, now common in the London area. Invasive species in the Thames estuary include the American slipper limpet, *Crepidula fornicata*, a filter feeder. This species comprises the largest biomass in the estuary. Densities of several thousand per square metre could be reached [112] under optimal conditions, and they are subject to mass die-off during cold weather as the Thames is towards the northerly limit of their range [113]. As a result, empty shells build drifts on beaches along the Kent coast and around the estuary, an indication that occurs in other invasive bivalves such as the Asiatic clam and zebra mussel in the Great Lakes in the United States. However, densities of *C. fornicata* in the UK remain low compared to those in warmer waters and may therefore be expected to increase. For comparison, the species is a problem in France, where populations are large and dense enough to reduce available habitat for flatfishes [114]. Dredging is performed to reduce density, but this may aggravate the problem through provision of additional habitat. The shells are used to supplement building materials [115]. It is not possible to conclude that native bivalve populations have been only negatively affected and driven out by the slipper limpet as most were in decline in many areas due to other problems, including invasive drill predation on oyster spat and Tributyltin (TBT) contamination from antifouling paint applied to boats and other structures. Studies indicate that native oysters occupy different areas of the benthos [116]. However, as filter feeders, the slipper limpet can cause a reduction in food availability. In relation to other species, *C. fornicata* can reduce star fish predation on the blue mussel, *Mytilus edulis* [117]. Epifaunal slipper limpets can promote increased byssal production in blue mussels, thereby redirecting resources away from growth and reproduction. They accumulate in reproductive stacks on top of the mussels, increasing stress on the byssal fibres anchoring them to the substrate, which can also trigger their detachment during storms.

An example of problems associated with non-native species can be illustrated with the American oyster, *Crassostrea virginica*. In the 1950s, this species aided the importation of an associated assemblage through ‘hitch-hiking’, where other species travelled attached to or inside the oysters. These included the oyster drill, *Urosalpinx cinereal*, and various pathogens that have led to outbreaks of disease and decline of European oysters, *Ostrea edulis*. Populations of the oyster drill reached densities of 15,000–20,000 per hectare in areas of Thames estuary and were believed to account for spat mortalities of up to 75%, with each drill capable of consuming 40 oysters per year. However, *U. cinereal* numbers declined along with oyster populations. They were further severely affected through imposex, the development of male gonads in females, caused by Tributyltin (TBT), leading to their rapid decline in the mid-twentieth century [118]. As with many banned chemicals, TBT persists in sediment and continues to be an important pollutant affecting aquatic species and human health [119].

Fossorial species are a concern as they can cause damage to exposed banks. Several species of crayfish now occur in the UK, including the native white-clawed variety and Turkish narrow-clawed crayfish. The brown rat, *Rattus norvegicus*, originally an invasive species from Asia, has been present in the UK since before 1800. It is very numerous in the Thames and in terms of damage is certainly one of the most destructive. Additional burrowing animals include the black rat, *Rattus rattus*, American mink, *Neovison vison,* and to a lesser extent, the Chinese mitten crab, *Eriocheir sinensis*. Invasions by *Eriocheir sinensis*, the Chinese mitten crab, occurred in northern Europe in the early 1900s and in California in the United States in the 1990s. The species has established viable populations in several countries, including the UK, where it has not been designated as invasive. Species invasions are frequently a symptom of wider environmental problems that initiate changes before settlement occurs and reduce resilience in the face of propagule pressure. The concept of non-native invasive species (NNIS) against a static, native flora and fauna needs to be re-evaluated in the face of climate change and accumulated degradation of waterbodies, as in its current form NNIS embodies many contradictions, which could impede efforts to maintain habitat and ecosystem resilience. For example, species range shifts are proceeding at an accelerating pace, i.e., native species are moving and there is increasing interest in translocations for conservation purposes, termed ‘assisted migration’. Additionally, the endemic flora and fauna of rivers worldwide now include a variety of NNIS that have become endemic over decades or centuries. A comprehensive ecosystem restoration framework over the entire warming event (perhaps several centuries) and its aftermath together with consideration of positive contributions from non-native species, such as their functional role, as well as side-effects of control methods (some of which are currently performed for purely cosmetic reasons) would improve resource allocation and planning. In the Thames, for example, non-native species, including the Chinese mitten crab and several species of fish and plants, such as Japanese knotweed and Himalayan balsam, will not easily be extirpated and are likely to remain as long as climate remains suitable, so more nuanced control methods are required.

## 4. Conclusions

This paper overviews anthropogenic changes to rivers in the urban environment, the so-called urban river syndrome, and discusses urban rivers’ deteriorating ecology and conservation challenges of river rejuvenation. Sustainable use of natural resources by humans will require transformation of many processes in order that they return a positive contribution to the environment. It will require movement towards a more sustainable, circular economy, one that recycles natural resources, otherwise conditions will worsen through continued accumulation of wastes.

Systems theory is frequently mentioned in relation to rivers, but particular aspects of systems integration are not widely appreciated, in particular, how general systems theory might elucidate phenomena such as phase change or abrupt, nonlinear decline. We believe, for example, that non-native species irruptions may be described in these terms as transient phenomena rather than permanent issues. 

Restoration projects addressing urban rivers and wider ecosystems will need to consider a broad range of scientific, social and economic factors at a wide range of spatial and temporal scales to maximise potential benefits. Non-native species, some of which are thriving in the face of environmental change, will need to be assessed for their potential contribution to ecosystem stability, particularly in the face of potential collapse. Restoration work will need to make a positive contribution towards sustainability, satisfy stakeholder wishes and achieve benefits for an appropriate period of time in relation to cost.

The current definition of the urban river syndrome is expanded to include other features of rivers, such as riparian habitat degradation, as an initial step in transforming the concept (as well as river restoration in general) to comply with the requirements of achieving sustainability. A small demonstration project has been conducted in the river Thames in the application of the revised definition. The work has been carried out as a transdisciplinary experiment with assistance from multiple commercial, academic and non-profit partners and charities with involvement of the local community. An assessment of riparian soil contamination, river bank stability and erosion was made with wider consideration of climate change, sea-level rise, pollution and other features highlighted in the revised URS.

In progressing towards a more holistic and therefore general definition, we hope eventually to incorporate or connect it with other elements of socio-ecological-technical systems (SETs) so that restoration work may be comprehensively assessed: it should be socially warranted (achieving outcomes desired by the community and stakeholders), conform with and value historical and cultural aspects, be economically justifiable, and the life-expectancy of the work must be explored. In this manner restoration will be guided by the multifarious interests of stakeholders and prioritised against hazards, existential risk, costs and benefits within a rational and forward-looking context.

## Figures and Tables

**Table 1:** Notable ecological theories.

Ecological Theories	Year Introduced	Recently Reviewed
The River Continuum Concept (RCC)	1980	[4]
Hierarchical Patch Dynamics (HPD)	1940s	[5]
Functional Process Zones (FPZ)	1980	[6]

**Table 2:** Additional and increasing symptoms of an updated urban river syndrome ^1^.

Aquatic & Riparian Biota		Recent Reports
Species range shifts	New	[36]
Accelerating non-native species establishment	New	[37]
Riparian vegetation degradation and die-back, tree disease, invasive plants, clearance by humans	New	[38,39,40]
Increased prevalence of pathogens, including viruses, bacteria, and parasites	New	[41]
Reductions in biodiversity because of fish stocking and farming	New	[42,43]
Behavioural changes due to domesticated transplants and xenobiotics	New	[44]
**Ecosystem processes**		
Migration between landfills and waste sites by gulls with increasing predation in rivers and estuaries	New	[45,46]
Cancers in aquatic animals: birds, fish, bivalves and mammals	New	[47]
Toxin bioaccumulation in prey species with trophic amplification (e.g., per- and polyfluoroalkyl substances, PFASs)	New	[48,49,50,51]
Changes in diet	New	[52,53]
Interference with sound, light and chemical signalling regimes	New	[54,55,56,57]
**Atmosphere, soils, water, and sediment chemistry**		
Global warming (e.g., warmer water) with marine heat waves in the estuary and ocean	Increasing	[58]
Anthroposphere	New	[59]
NO_2_, CH_4_ and CO_2_ emissions from water bodies, landfills and sewage treatment plants (STPs)	New	[60]
Rubbish, including micro- and nanoplastics. Accumulation in animals, soils and the substrate with transport to the ocean	New	[61,62]
**Hydrology**		
Increasing extreme flooding and drought events	Increasing	[63]
**Channel morphology**		
Soil erosion with silt banks forming in the lower reaches	Increasing	[64]

^1^ Previously, the urban river syndrome has emphasised changes to river morphology and water chemistry but riparian habitat, soils or related fauna and flora were not included. In order to make the concept more general, these are added here together with disease. Further work is needed to incorporate extreme events such as flooding and landslides and combinations of different ecosystem processes to facilitate comprehensive assessment of condition.

**Table 3:** Emerging tree diseases, some of which are affecting riparian vegetation in the UK.

Condition	Causative Agent
Root and collar rot in alder	*Phytophthora alni* (Phytopthora)
Canker stain of plane trees	*Ceratocystis platani* (fungus)
Acute oak decline	Multiple environmental causes (e.g., pollution and climate change)
Horse chestnut leaf miner	*Cameraria ohridella* (moth)
Chalara die-back of Ash	*Hymenoscyphus fraxineus* (fungus)
Massaria disease	*Splanchnonema platani* (fungus)
Oak processionary moth	*Thaumetopoea processionea* (moth)
